# Supplementary material for: Revealing the relationship between liquid fragility and medium-range order in silicate glasses
Source: Nat Commun. 2023 Jan 3;14:13. doi: 10.1038/s41467-022-35711-6 (PMC9810649; doi:10.1038/s41467-022-35711-6)
Supplement: Supplementary file 1 — Supplementary Information [file 41467_2022_35711_MOESM1_ESM.pdf]

Supplementary Tables for

Revealing the Relationship between Liquid Fragility and Medium-Range Order in Silicate Glasses

Ying Shi,[*] Binghui Deng, Ozgur Gulbiten, Mathieu Bauchy, Qi Zhou, Jörg Neuefeind, Stephen R. Elliott, Nick J. Smith, Douglas C. Allan

*Corresponding author. e-mail: shiy3@corning.com

Table 1. Composition, *NBO/T* ratio, measurement-fitting equation and value of fragility, calculated *MRD* of 48 CAS glasses

<table>
<tr><th rowspan="2">Series</th><th colspan="3">Composition (mol%)</th><th rowspan="2">NBO/T</th><th colspan="4">Fragility</th><th rowspan="2">Calculated MRD* (Å)</th></tr>
<tr><th>SiO_2</th><th>Al_2O_3</th><th>CaO</th><th>Measurement method</th><th>Reported</th><th>Conversion or correction method</th><th>m-value</th></tr>
<tr><td rowspan="5">NBO/T=1 [1]</td><td>49.4</td><td>8.3</td><td>42.3</td><td>1.03</td><td rowspan="13">Dilatometry for high viscosity & viscometry for low viscosity</td><td>4.18</td><td rowspan="13">α-value from AV fit, m=13.7xα</td><td>57.27</td><td>3.49</td></tr>
<tr><td>45.6</td><td>10.5</td><td>43.9</td><td>1.00</td><td>4.36</td><td>59.73</td><td>3.46</td></tr>
<tr><td>41.3</td><td>12.7</td><td>46.1</td><td>1.00</td><td>4.4</td><td>60.28</td><td>3.41</td></tr>
<tr><td>37.3</td><td>14.7</td><td>47.9</td><td>1.00</td><td>4.61</td><td>63.16</td><td>3.37</td></tr>
<tr><td>33</td><td>16.4</td><td>50.5</td><td>1.04</td><td>4.62</td><td>63.29</td><td>3.32</td></tr>
<tr><td rowspan="5">NBO/T=0.5 [1]</td><td>49.8</td><td>15.1</td><td>35.1</td><td>0.50</td><td>3.83</td><td>52.47</td><td>3.50</td></tr>
<tr><td>44.1</td><td>17.4</td><td>38.5</td><td>0.53</td><td>4.05</td><td>55.49</td><td>3.44</td></tr>
<tr><td>39.5</td><td>19.9</td><td>40.5</td><td>0.52</td><td>4.25</td><td>58.23</td><td>3.39</td></tr>
<tr><td>34.8</td><td>22.5</td><td>42.7</td><td>0.51</td><td>4.4</td><td>60.28</td><td>3.34</td></tr>
<tr><td>29.4</td><td>24.7</td><td>45.9</td><td>0.54</td><td>4.56</td><td>62.47</td><td>3.29</td></tr>
<tr><td>Wollastonite (Wo) [2]</td><td>49.6</td><td>0.1</td><td>50.3</td><td>2.02</td><td>4.76</td><td>65.21</td><td>3.50</td></tr>
<tr><td>Anorthite (An) [2]</td><td>49.6</td><td>24.8</td><td>25.6</td><td>0.02</td><td>3.88</td><td>53.16</td><td>3.50</td></tr>
<tr><td>Gehlenite (Geh) [2]</td><td>24.4</td><td>25.1</td><td>50.5</td><td>0.68</td><td>4.86</td><td>66.58</td><td>3.23</td></tr>
<tr><td rowspan="4">NBO/T=0 this study</td><td>40.32</td><td>29.82</td><td>29.86</td><td>0.00</td><td rowspan="4">Isothermal equilibrium viscosity measurement near T_g</td><td>53.6</td><td rowspan="4">Linear fit of Angell plot, no correction</td><td>53.6</td><td>3.43</td></tr>
<tr><td>49.61</td><td>25.13</td><td>25.27</td><td>0.00</td><td>50.1</td><td>50.1</td><td>3.50</td></tr>
<tr><td>60.3</td><td>19.9</td><td>19.79</td><td>0.00</td><td>43.9</td><td>43.9</td><td>3.61</td></tr>
<tr><td>70.48</td><td>14.82</td><td>14.7</td><td>0.00</td><td>40.5</td><td>40.5</td><td>3.71</td></tr>
<tr><td rowspan="4">NBO/T=0 [3]</td><td>69</td><td>15.9</td><td>14.8</td><td>-0.02</td><td rowspan="6">Dilatometry (BBV, BPV & PPV) for high viscosity & viscometry (rotational)</td><td>38</td><td rowspan="6">m-value from MYEGA fit, no correction</td><td>38</td><td>3.70</td></tr>
<tr><td>54.9</td><td>22.6</td><td>22</td><td>-0.01</td><td>49</td><td>49</td><td>3.55</td></tr>
<tr><td>46.4</td><td>26.7</td><td>26.3</td><td>-0.01</td><td>54</td><td>54</td><td>3.46</td></tr>
<tr><td>30</td><td>35</td><td>34.3</td><td>-0.01</td><td>57</td><td>57</td><td>3.29</td></tr>
<tr><td rowspan="2">Constant CaO [3]</td><td>69.6</td><td>9.9</td><td>20</td><td>0.23</td><td>41</td><td>41</td><td>3.71</td></tr>
<tr><td>66.9</td><td>12.3</td><td>20.3</td><td>0.17</td><td>44</td><td>44</td><td>3.68</td></tr>
</table>

	64.4	15	20.2	0.11	for low viscosity	46		46	3.65
	62.8	17.3	19.5	0.05		45		45	3.63
	60.3	19.7	19.6	0.00		46		46	3.61
	58.5	22.1	19	-0.06		51		51	3.59
	55.3	24.6	19.6	-0.10		48		48	3.56
	52.7	26.8	20.1	-0.13		48		48	3.53
**Eutectic** [4]	64.9	9.3	25.8	0.40	Dilatometry for high viscosity & viscometry for low viscosity	3.74	*F*-value from AM fit, *m*= (12-log η_∞) x *F*	47.5	3.66
	59.9	10	30.1	0.50		3.7		48	3.60
	54.7	10.9	34.4	0.61		3.7		50	3.55
	49.6	11.7	38.7	0.74		3.8		52	3.50
	44.4	12.5	43.1	0.88		3.8		53	3.44
**Non-eutectic** [4]	61.6	14.9	23.4	0.19		2.9		40	3.62
	57.8	15.8	26.3	0.23		3.2		44	3.58
	51.9	16.8	31.3	0.34		3.6		48	3.52
	48.1	18.9	33	0.33		3.69		51	3.48
	42.7	19.3	38	0.46		3.4		49	3.42
**Constant SiO_2** [5]	67	19.73	13.2	-0.12	DSC	40	Corrected by Zheng's equation [6]	47.2	3.68
	67.8	17.68	14.5	-0.06		38		44.7	3.69
	67.4	16.28	16.3	0.00		44		52.4	3.68
	68.7	13.99	17.3	0.07		40		47.2	3.70
	68	13.08	18.9	0.12		37		43.4	3.69
	67.4	10.32	22.3	0.27		41		48.5	3.68
	65.7	9.06	25.3	0.39		41		48.5	3.66
	66.7	6.65	26.6	0.50		45		53.7	3.67
	64.5	5.49	30	0.65		46		55.0	3.65

* *MRD* values are calculated by the linear equation, *MRD* = 3.01 + 0.01 x SiO_2 (mol%), which is derived from the 27 neutron-measured CAS glasses listed in Table 2 below and plotted in Fig.2 (a) of the main text.

Table 2. Composition, *NBO/T* ratio, RingFSDP deconvolution analyses of 27 CAS glasses

Composition (mol%)			*NBO/T*	RingFSDP fitting			
SiO_2	Al_2O_3	CaO		$f_{\leq 4ring}$	f_{5ring}	$f_{\geq 6ring}$	*MRD (Å)*
50.37	9.64	39.99	0.87	0.58	0.36	0.07	3.42
41.53	19.38	39.09	0.49	0.60	0.32	0.08	3.42
59.60	9.91	30.49	0.52	0.30	0.70	0.00	3.53
49.62	19.77	30.61	0.24	0.39	0.61	0.00	3.49
40.96	28.99	30.04	0.02	0.48	0.52	0.00	3.43
50.28	24.46	25.26	0.02	0.35	0.65	0.00	3.51
69.93	9.84	20.23	0.23	0.24	0.53	0.23	3.71
65.13	14.71	20.16	0.12	0.23	0.64	0.13	3.65
60.06	19.84	20.10	0.01	0.21	0.79	0.00	3.59
75.05	5.01	19.94	0.35	0.21	0.47	0.32	3.77
65.40	17.45	17.15	-0.01	0.23	0.66	0.11	3.63
65.40	17.45	17.15	-0.01	0.23	0.62	0.15	3.66
69.87	14.93	15.20	0.01	0.23	0.54	0.23	3.72
79.77	5.09	15.15	0.22	0.18	0.44	0.38	3.82
65.09	19.85	15.06	-0.09	0.23	0.59	0.17	3.67
74.95	10.10	14.94	0.10	0.20	0.48	0.32	3.78
74.96	14.98	10.06	-0.09	0.20	0.51	0.30	3.77
85.02	4.97	10.00	0.11	0.14	0.40	0.46	3.90
80.35	9.76	9.89	0.00	0.16	0.46	0.38	3.84
63.77	18.06	18.17	0.00	0.24	0.66	0.10	3.63
67.00	16.66	16.34	-0.01	0.24	0.57	0.18	3.67
74.87	12.29	12.85	0.01	0.20	0.49	0.31	3.77
40.32	29.82	29.86	0.00	0.41	0.59	0.00	3.47
49.61	25.13	25.27	0.00	0.33	0.67	0.00	3.52
60.3	19.9	19.79	0.00	0.18	0.82	0.00	3.60
70.48	14.82	14.7	0.00	0.20	0.54	0.25	3.74
100.00	0.00	0.00	0.00	0.12	0.31	0.57	3.98

Table 3. Composition, *NBO*/*T* ratio, measurement-fitting equation and value of fragility, neutron-measured *MRD* of 20 NAS glasses with Mg or Ca

Series	Composition (mol%)				NBO/T	Fragility					Combined Fragility		Measured *MRD* (Å)
	SiO_2	Al_2O_3	Na_2O	MgO /CaO		Measurement method	Fitting method	*m*-value	Fitting method	*m*-value	Value	Error	
Mg-NaAS	75.83	0.07	15.63	8.11	0.62	Dilatometry (BBV & PPV) for T_{anneal} & T_{soft}, viscometry (rotational) for low viscosity	MYEGA fit [7]	31.3	Modified elastic model [8]	29.7	30.5	1.1	3.75
	73.7	2.71	15.73	7.62	0.52			32.6			32.6		3.75
	70.88	5.32	15.68	7.88	0.45			32.7			32.7		3.74
	68.07	7.99	15.71	7.98	0.37			32.9		31.5	32.2	1.0	3.72
	65.33	10.72	15.74	7.95	0.30			34			34		3.72
	62.77	13.31	15.78	7.9	0.23			33.6			33.6		3.71
	59.92	15.98	15.77	8.08	0.17			34.4		33.2	33.8	0.8	3.69
	56.62	18.63	15.55	8.94	0.12			35.9			35.9		3.68
	54.64	21.33	15.78	7.99	0.05			38.7			38.7		3.66
	52.02	23.97	15.82	7.93	0.00			39.6		38.6	39.6	0.7	3.64
Ca-NaAS	75.83	0.07	15.63	8.11	0.62			37.9		35.6	36.75	1.6	3.59
	73.7	2.71	15.73	7.62	0.52			37.7			37.7		3.62
	70.88	5.32	15.68	7.88	0.45			37			37		3.63
	68.07	7.99	15.71	7.98	0.37			38.4		35.3	36.85	2.2	3.62
	65.33	10.72	15.74	7.95	0.30			38.4			38.4		3.62
	62.77	13.31	15.78	7.9	0.23			37.4			37.4		3.63
	59.92	15.98	15.77	8.08	0.17			36.6		35.2	35.9	1.0	3.65
	56.62	18.63	15.55	8.94	0.12			36.9			36.9		3.66
	54.64	21.33	15.78	7.99	0.05			38.9			38.9		3.66
	52.02	23.97	15.82	7.93	0.00			40.8		39.8	40.3	0.7	3.63

Table 4. Composition, *NBO*/*T* ratio, RingFSDP deconvolution analyses of five NAS and one NS glasses

Composition (mol%)			*NBO/T*	RingFSDP fitting			
SiO_2	Al_2O_3	Na_2O		$f_{\leq 4ring}$	f_{5ring}	$f_{\geq 6ring}$	*MRD (Å)*
65.69	14.84	19.47	0.10	0.28	0.38	0.35	3.76
64.01	18.13	17.86	-0.01	0.23	0.38	0.40	3.81
70.66	9.95	19.39	0.21	0.29	0.36	0.35	3.75
70.29	14.70	15.01	0.01	0.20	0.39	0.41	3.84
75.58	12.41	12.00	-0.01	0.18	0.40	0.42	3.85
80.30	0.00	19.70	0.49	0.29	0.35	0.36	3.79

Table 5. Composition, *NBO/T* ratio, measurement-fitting equation and value of fragility, calculated or neutron-measured *MRD* of 14 NAS glasses

**Series**	**Composition (mol%)**			**NBO/T**	**Fragility**				**Calculated *MRD** (Å)**
	**SiO_2**	**Al_2O_3**	**Na_2O**		**Measurement method**	**Reported**	**Conversion or correction method**	***m*-value**	
**Constant SiO_2** [5]	68.5	20.1	11.4	-0.16	DSC	29	Corrected by Zheng's equation $m_{vis} = 1.29m_{DSC} - 4.33$ [6]	33.1	3.91
	68.2	19	12.8	-0.12		28		31.8	3.90
	66.9	19.8	13.4	-0.12		28		31.8	3.90
	67.6	17.1	15.3	-0.04		27		30.5	3.86
	67.2	16.86	16	-0.02		28		31.8	3.85
	65	17.84	17.2	-0.01		29		33.1	3.85
	66.7	16.35	17	0.01		25		27.9	3.84
	66.6	15.5	17.89	0.05		30		34.4	3.82
	69.9	12.7	17.38	0.10		30		34.4	3.80
	67.5	13.2	19.3	0.13		31		35.6	3.78
	67.3	11.7	20.99	0.20		32		36.9	3.75
	67.3	9.8	22.93	0.30		32		36.9	3.70
	68.4	1.73	30.15	0.79		40		47.2	3.48
**NBO/T=0 (Albite)** [8]	75	12.5	12.5	0.00	Dilatometry (BBV & PPV) for T_{anneal} & T_{soft}, viscometry (rotational) for low viscosity	25.6	Modified elastic model [7]	25.6	$3.85^{mea.}$

* *MRD* values are calculated by the linear equation, *MRD* = 3.82 - 0.50 x NBO/T, averaged from the two linear lines derived from 65 (solid blue) and 70 (dotted blue) mol% SiO_2 NAS glasses, as shown in Fig. 4e of the main text.

Table 6. Description, *NBO*/*T* ratio, measurement-fitting equation-value of fragility, neutron-measured *MRD* of 4 *in-situ* glasses and two fictitious glasses

Series	Description	Fragility			NBO/T	Measured *MRD* (Å)
		Measurement method	Fitting method	*m*-value		
**Jade** [9]	Mixed alkaline earth aluminosilicate	Dilatometry (BBV & PPV) for T_{anneal} & T_{soft}, viscometry (rotational) for low viscosity	Modified elastic model [8]	35	-0.04	3.84
**Jade** [9]	Mixed alkaline earth aluminosilicate	Dilatometry (BBV & PPV) for T_{anneal} & T_{soft}, viscometry (rotational) for low viscosity	MYEGA fit [10]	36.8	-0.04	3.84
**Jade** [9]	Mixed alkaline earth aluminosilicate	Isothermal equilibrium viscosity measurement near T_g	Linear fit	32.4	-0.04	3.84
**Jade** [9]	Mixed alkaline earth aluminosilicate	**Average**		**34.7**	-0.04	3.84
**Jade** [9]	Mixed alkaline earth aluminosilicate	**Std. Dev.**		**2.2**	-0.04	3.84
**NIST 710a** [11]	Soda-lime silica	Dilatometry (BBV & PPV) for T_{anneal} & T_{soft}, viscometry (rotational) for low viscosity	Modified elastic model [8]	30.3	0.62	3.93
**NIST 710a** [11]	Soda-lime silica	Isothermal equilibrium viscosity measurement near T_g	Linear fit	34.9	0.62	3.93
**NIST 710a** [11]	Soda-lime silica	**Average**		**33.1**	0.62	3.93
**NIST 710a** [11]	Soda-lime silica	**Std. Dev.**		**4.0**	0.62	3.93
**CG** [12]	Mixed alkaline aluminosilicate	Isothermal equilibrium viscosity measurement near T_g	Linear fit	29.1	0.08	4.01
**FS**	Glassy SiO_2	Dilatometry for high viscosity & viscometry for low viscosity [13] & DSC	MYEGA fit [14]	20	0	4.13
**Fictitious$_{low}$**	Lowest-*m* glass with only ≥6-membered rings			14.93		4.3
**Fictitious$_{high}$**	Highest-*m* glass with only ≤4-membered rings			69.56		3.15

Table 7. Composition, T_g, fragility, RT-*MRD*, the slope of *in-situ MRD* with *T* change for $slope_g$, $slope_l$, and their ratio ($slope_l/slope_g$). The error in the slope for the *S*(*Q*)-FSDP area and *MRD* change is determined from linear fitting. The errors for the liquid/glass ratio for *MRD* slopes are calculated by error propagation

Glass I.D.	Composition (mol%)			T_g (K)	Fragility*	RT-*MRD* (Å)	Slope of in-situ *MRD* with *T*		
	CaO or Na_2O	Al_2O_3	SiO_2				$Slope_{glass}$	$Slope_{liquid}$	Slope Ratio (liquid/glass)
**CAS40**	29.86	29.82	40.32	1126.7	53.6(8)	3.47	0.029(2)	0.124(3)	4.2(1)
**CAS50**	25.27	25.13	49.61	1132.8	50.1(9)	3.52	0.028(1)	0.111(1)	3.90(6)
**CAS70**	14.70	14.82	70.48	1148.1	40.5(6)	3.74	0.027(2)	0.063(3)	2.38(9)
**NS20**	20.50	0.00	79.50	756.2	37.0	3.79	0.020(2)	0.030(4)	1.5(1)

*The fragility of three CAS glasses were measured by an isothermal equilibrium viscosity method in this study (see Supplementary Note), NS20 fragility is from Ref. [15].

Table 8. *MRD* (Å) derived from *in-situ* neutron FSDP of three CAS and NS20 glasses

T/T_g	CAS40	CAS50	CAS70	NS20
0.26	3.499	3.546	3.741	
0.40	3.510	3.555		3.769
0.50	3.515	3.571	3.757	3.777
0.60	3.523	3.577		3.784
0.70	3.538	3.590	3.779	3.793
0.80	3.547	3.599		3.797
0.90	3.563	3.608	3.802	3.802
1.00	3.577	3.622	3.815	3.822
1.05	3.608	3.638	3.826	3.829
1.10	3.621	3.662	3.839	3.836
1.15				3.835
1.20				3.848
1.25				3.850

References

[1] M. Solvang, Y.Z. Yue, S.L. Jensen, D.B. Dingwell, "Rheological and thermodynamic behaviors of different calcium aluminosilicate melts with the same non-bridging oxygen content," *J. Non-Cryst. Solids,* vol. 336, pp. 179-188, 2004.

[2] M. Solvang, Y.-Z. Yue, S.L. Jensen, D.B. Dingwell, "Rheological and thermodynamic behavior of calcium aluminosilicate melts within the anorthite– wollastonite–gehlenite compatibility triangle," *J. Non-Cryst. Solids,* vol. 351, pp. 499-507, 2005.

[3] T.K. Bechgaard, J.C. Mauro, M. Bauchy, Y. Yue, L.A. Lamberson, L.R. Jensen, M.M. Smedskjaer, "Fragility and configurational heat capacity of calcium aluminosilicate glass-forming liquids," *J. Non-Cryst. Solids,* vol. 461, pp. 24-34, 2017.

[4] M. Moesgaard, Y. Yue, "Compositional dependence of fragility and glass forming ability of calcium aluminosilicate melts," *J. Non-Cryst. Solids,* vol. 355, pp. 867-873, 2009.

[5] S. L. Webb, "Configurational heat capacity of Na_2O–CaO–Al_2O_3–SiO_2 melts," *Chem. Geol.,* vol. 256, pp. 92-101, 2008.

[6] Q. Zheng, J.C. Mauro, Y. Yue, "Reconciling calorimetric and kinetic fragilities of glass-forming liquids," *J. Non-Cryst. Solids,* vol. 456, pp. 95-100, 2017.

[7] M.M. Smedskjaer, J.C. Mauro, J. Kjeldsen, Y. Yue, "Microscopic origins of compositional trends in aluminosilicate glass properties," *J. Am. Ceram. Soc.,* vol. 96, no. 5, pp. 1436-1443, 2013.

[8] S.P. Jaccani, O. Gulbiten, D.C. Allan, J.C. Mauro, Li. Huang, "Modified elastic model for viscosity in glass-forming systems," *Phys. Rev. B,* vol. 96, p. 224201, 2017.

[9] M. Potuzak, J. C. Mauro, T. J. Kiczenski, A. J. Ellison, D. C. Allan, "Communication: Resolving the vibrational and configurational contributions to thermal expansion in isobaric glass-forming systems," *J. Chem. Phys.,* vol. 133, pp. 091102-1-4, 2010.

[10] X. Guo, J.C. Mauro, D.C. Allan, M.M. Smedskjaer, "Predictive model for the composition dependence of glassy dynamic," *J. Am. Ceram. Soc.,* vol. 101, pp. 1169-1179, 2018.

[11] "Certificate for Standard Reference Material 710a, Soda-lime silica Glass," National Institute of Standards and Technology, Gaithersburg, MD 20899, USA , 1991.

[12] R.C. Welch, J.R. Smith, M. Potuzak, X. Guo, B.F. Bowden, T.J. Kiczenski, D.C. Allan, E.A. King, A.J. Ellison, J.C. Mauro, "Dynamics of glass relaxation at room temperature," *Phys. Rev. Lett.,* vol. 110, pp. 265901-1-4, 2013.

[13] G. Urbain, Y. Bottinga, P. Richet , "Viscosity of liquid silica, silicates and alumino-silicates," *Geochim. Cosmochim. Acta,* vol. 46, pp. 1061-1072, 1982.

[14] Y. Yue, "Anomalous enthalpy relaxation in vitreous silica," *Front. Mater,* vol. 2, pp. 54-1-11, 2015.

[15] R. Bohmer, K.L. Ngai, C.A. Angell, D.J. Plazek, "Nonexponential relaxations in strong and fragile glass formers," *J. Chem. Phys.,* vol. 99, pp. 4201-4209, 1993.

Supplementary Notes for

Revealing the Relationship between Liquid Fragility and Medium-Range Order in Silicate Glasses

Ying Shi,[*] Binghui Deng, Ozgur Gulbiten, Mathieu Bauchy, Qi Zhou, Jörg Neuefeind, Stephen R. Elliott, Nick J. Smith, Douglas C. Allan

*Corresponding author. e-mail: shiy3@corning.com

1. Linear *MRD* – SiO_2 mol% correlation of 27 CAS glasses

Room-temperature (RT) neutron total-scattering patterns were collected for 27 CAS glasses, with their XRF-analyzed compositions being listed in Supplementary Table 2. There are many ways to group these 27 glasses and search for correlations between structural parameters and SiO_2 mol% content. Here, we explore the evolution of the *MRD* structural parameter along both vertical and horizontal joins in the CAS ternary phase diagram.

MRD of vertical compositional line with constant NBO/T

As shown in Fig. 1a, five vertical lines are drawn in the CAS phase diagram. This is motivated by the fact that the glass compositions on each vertical join feature a constant *NBO/T* value but different SiO_2 mol% values. The *NBO/T* ratio is here defined as the amount of non-bridging oxygen (*NBO*) per glass-former tetrahedron (*T*) and is calculated by:

$$NBO/T = \frac{CaO\ mol\% \times 2 - Al_2O_3\ mol\% \times 2}{Al_2O_3\ mol\% \times 2 + SiO_2\ mol\%} \quad (1)$$

for CAS glasses. It is noted that two glasses contain more Al_2O_3 than CaO, which results in negative values of *NBO*/*T* according to the above equation. Strictly speaking, negative values of the *NBO*/*T* ratio are not physically meaningful but indicate a deficit of network modifiers to charge-compensated AlO_4 units — thereby resulting in the formation of higher-coordinated Al units or tricluster oxygen atoms.

The FSDPs of the neutron structure factor of the glasses on each vertical compositional line are plotted together. Five plots associated with *NBO/T* values ranging from 0.50 to -0.1 are shown in Fig. 1, b to f, respectively. In each plot, the *F*(*Q*)-FSDPs are labeled by their SiO_2 mol% compositions. All the five plots show that the FSDP position systematically shifts to the lower-*Q* range as the SiO_2 mol% increases, which corresponds to an increase in *MRD* values.

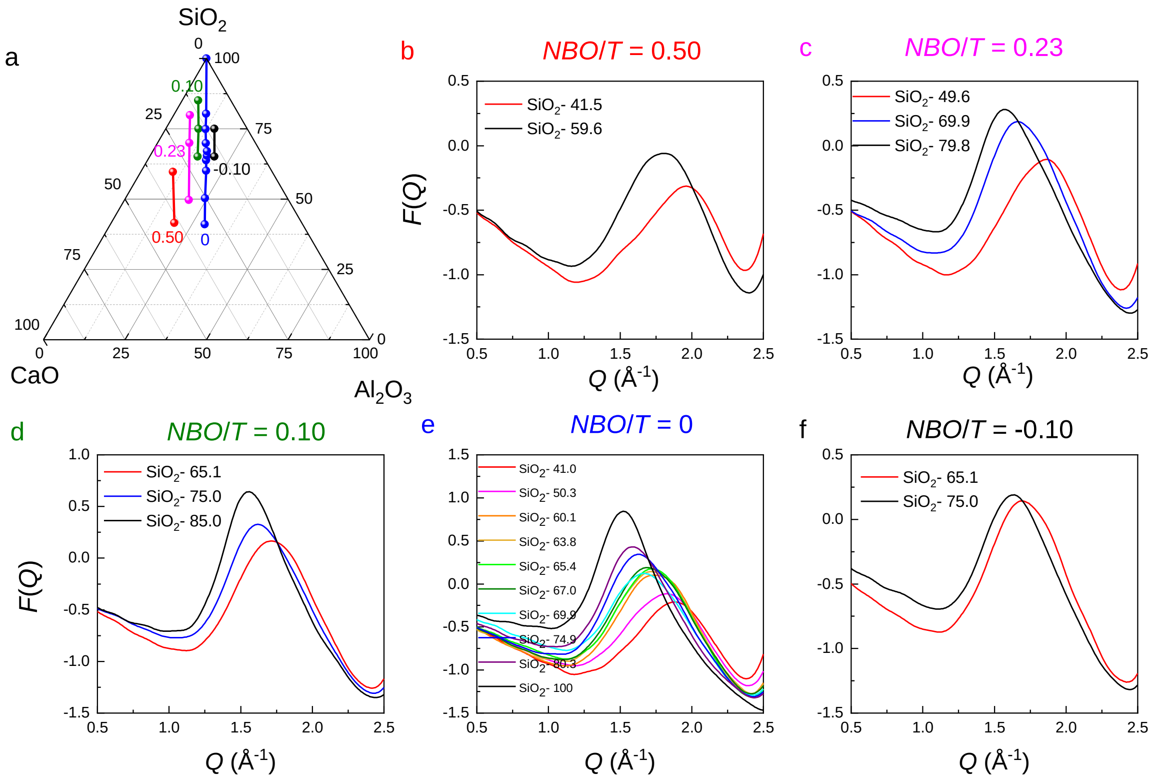

Fig. 1 | *MRD* value increases with increase in the SiO_2 mol% for glasses with compositions on the same vertical compositional line. Five vertical compositional lines within the CAS ternary phase diagram (**a**), with each line representing compositions with the same *NBO/T* ratio but different SiO_2 mol% values. The room-temperature neutron *F*(*Q*)-FSDPs of compositions along the five lines are plotted in (**b**) to (**f**), respectively, with their *NBO*/*T* values specified at the top. In each plot, the *F*(*Q*)-FSDPs of glasses, labeled by their SiO_2 mol% values, shift toward lower-*Q* values as the SiO_2 mol% content increases, indicating an increase of *MRD*.

MRD of horizontal compositional lines with constant SiO_2 content (mol%)

Seven horizontal lines are drawn in the CAS phase diagram (Fig. 2a). The glass compositions on each join have the same SiO_2 mol% values but different *NBO/T* ratios. Seven plots with SiO_2 mol% values ranging from 40 to 80 are shown in Fig. 2, b to h, respectively. In each plot, the *F*(*Q*)-FSDPs are labeled by their *NBO/T* values. For the glass groups with SiO_2 mol% ≥ 65 (Fig. 2, e to h), all the FSDPs in each group show the same position, regardless of their different *NBO/T* values. For the glass groups with SiO_2 mol% ≤ 60 (Fig. 2, b to d), the glass with the highest *NBO/T* value in each group shifts to a higher-*Q* value, corresponding to a smaller *MRD* value.

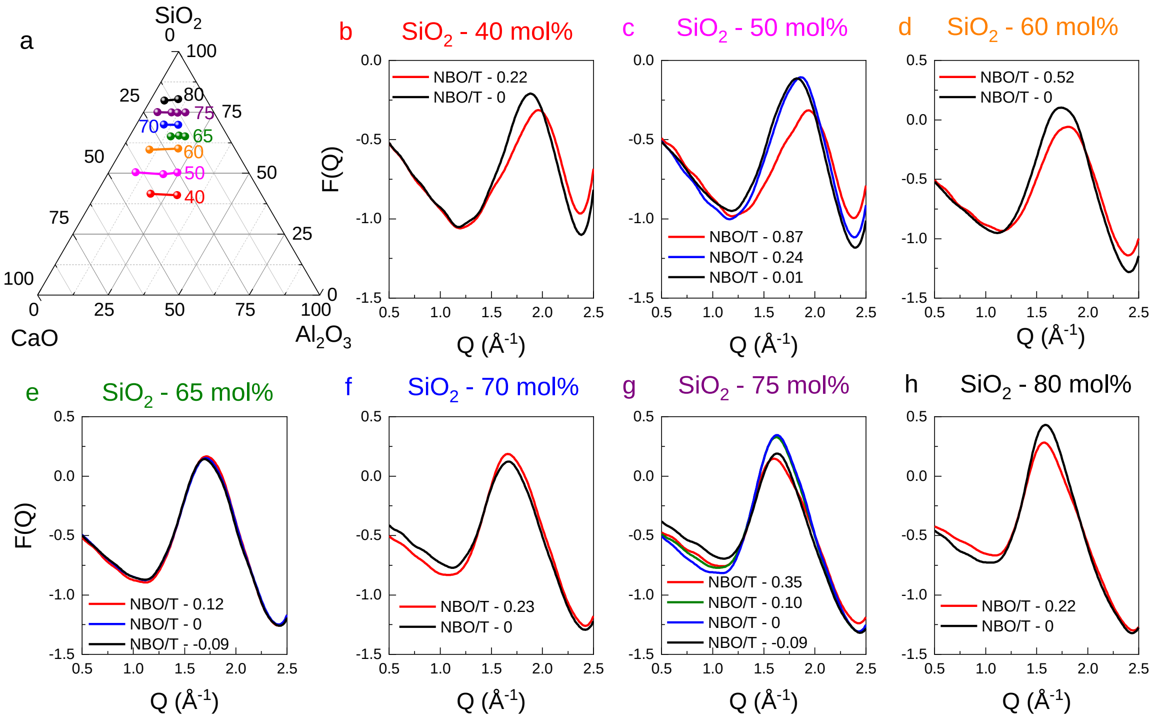

Fig. 2 | Same *MRD* value for glasses with compositions on the same horizontal compositional line. Seven horizontal lines within the CAS ternary phase diagram (**a**), with each line representing compositions with the same SiO_2 mol% values but different *NBO*/*T* ratios. The room-temperature neutron *F*(*Q*)-FSDPs of glasses with compositions within each horizontal line are plotted together. Seven groups of glasses with SiO_2 mol% values ranging from 40 to 80 are plotted in (**b**) to (**h**), respectively. In each plot, glasses with the same SiO_2 mol% values but different *NBO*/*T* ratios show no significant FSDP-position difference, except for the glass with the highest *NBO*/*T* ratio, in plots (**b**), (**c**) & (**d**), indicating that the majority of glasses with the same SiO_2 mol% value have the same *MRD* value.

2. No inverse correlation between *m*-SiO_2 mol% for the non-CAS glasses

To further confirm that *m* depends on *MRD* but not on SiO_2 mol%, we plot both the *m*-*MRD* and *m*-SiO_2 mol% correlations in Fig. 3**Error! Reference source not found.** below. In panel (a), an inverse correlation between *m* and *MRD* with R^2=0.77 is observed for all 88 glasses. In panel (b), an inverse correlation between *m* and SiO_2 mol% is also found for all 88 glasses, with a fair coefficient of determination R^2 = 0.60. However, we argue that this apparent correlation mainly originates from the 48 CAS glasses. We then remove the 48 CAS glasses and only plot the *m*-*MRD* and *m*-SiO_2 mol% correlations for the remaining 40 glasses in panels (c) and (d). In panel (c), an inverse correlation of *m* with *MRD* still holds valid, with a slightly lower R^2 value of 0.61. In contrast, no correlation of *m* and SiO_2 mol% is observed for the remaining 40 glasses in panel (d). This confirms that *m* does not depend on SiO_2 mol% for silicate glasses, except for the CAS system.

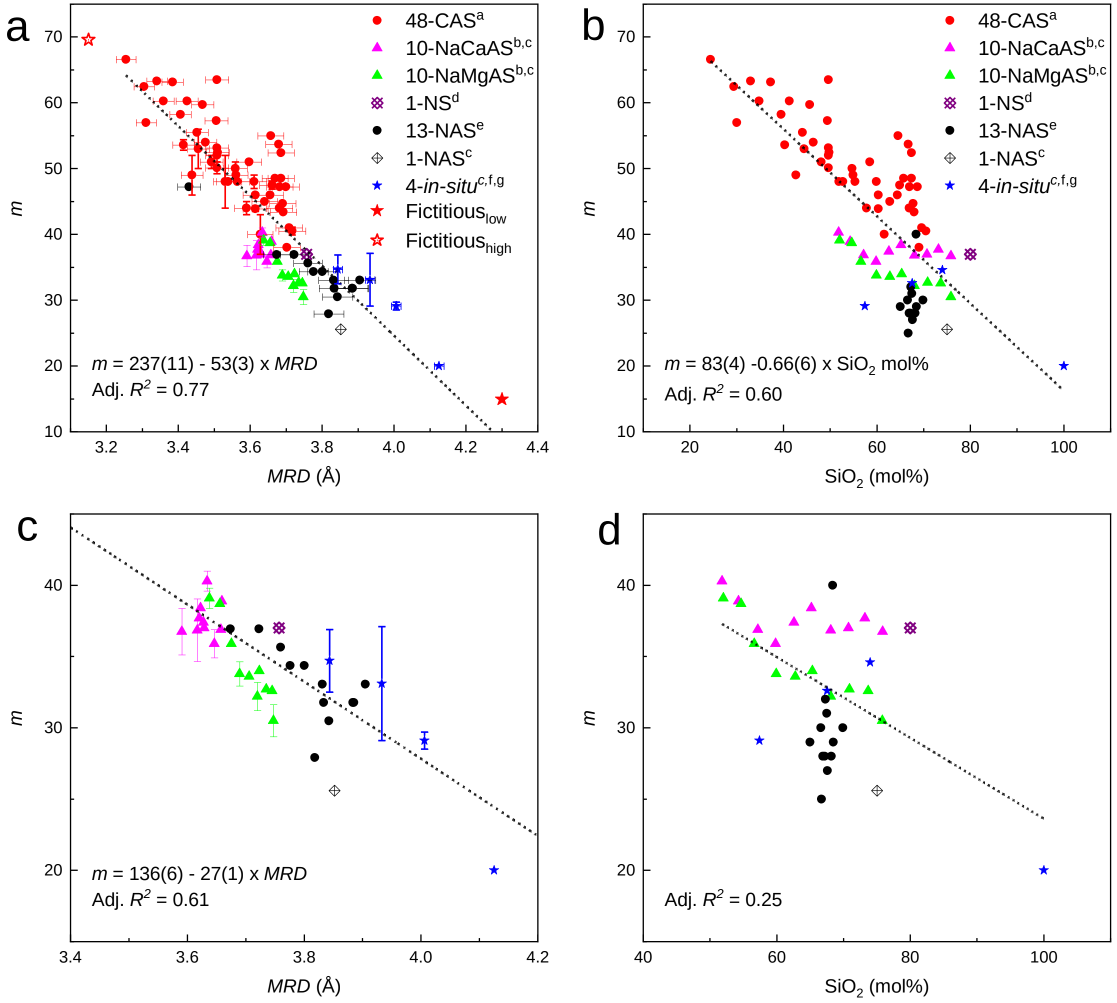

Fig. 3 | Comparison of the *m-MRD* and *m*-SiO_2 mol% correlations. (a) Inverse correlation between *m* and *MRD* with R^2 = 0.77 for all the 88 glasses. (b) Inverse correlation between *m* and SiO_2 mol% with R^2 = 0.60 for all the 88 glasses. (c) Inverse correlation between *m* and *MRD* with R^2 = 0.61 for 40 glasses, with the 48 CAS glasses being removed. (d) No correlation between *m* and SiO_2 mol% with R^2 = 0.25 for all 40 glasses, with the 48 CAS glasses being removed.

3. CAS fragility measurement by an isothermal equilibrium viscosity method

Equilibrium three-point beam-bending viscosity measurements were conducted for four charge-balanced CAS glasses xCaO–$x$$Al_2O_3$–(1-2$x$)$SiO_2$ (x = 0.15, 0.2, 0.25, 0.3) (the nomenclature of the CAS glasses is given by the SiO_2 content, i.e., the glass with x = 0.15 is designated as CAS70). Two of those glasses, CAS40 and CAS50, were measured twice for two separate sample preparations. The T_g-scaled Arrhenius plots of the viscosity of the six measurements are shown in Fig. 4. Employing Angell's definition of the glass transition and fragility index, the fragility-index values were calculated and are listed in Fig. 4. The fragility-index error of CAS 60 and 70 glasses, which were measured once, is determined from the error of linear fitting of each Arrhenius plot. The errors for CAS 40 and 50, which have two measurements, are propagated from the errors of two independent linear fittings.

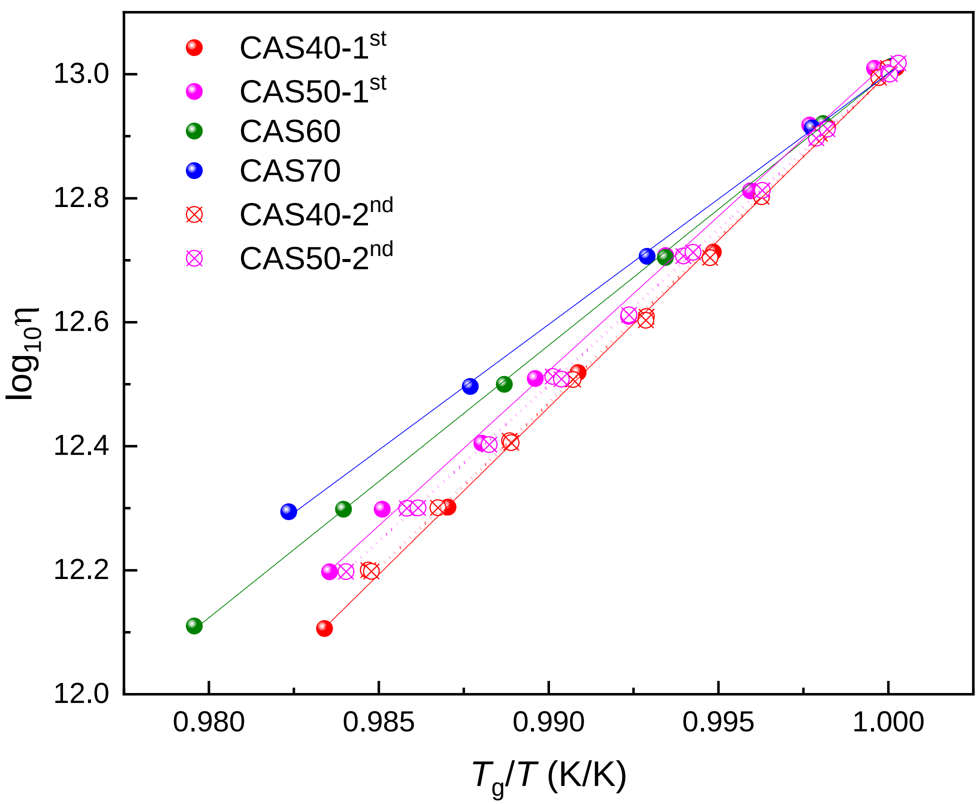

Fig. 4 | T_g-scaled Arrhenius plot of the viscosity of four CAS glasses. Glasses CAS40 and CAS50 were measured twice for two individual sample preparations to test the measurement reproducibility.

Table 1 . Composition, T_g, and fragility of four CAS glasses. The fragility error is derived from the error of the linear fit.

Glass ID.	Composition (mol%)			Mea.	T_g (K)	Fragility	
	SiO_2	Al_2O_3	CaO			Value	Error
**CAS40**	40.32	29.82	29.86	1st	1127.0	53.9	0.6
				2nd	1126.5	53.2	0.5
				Ave.	1126.7	53.6	0.8
**CAS50**	49.61	25.13	25.27	1st	1132.8	49.9	0.9
				2nd	1132.8	50.3	0.3
				Ave.	1132.8	50.1	0.9
**CAS60**	60.3	19.9	19.79	1st	1137.4	43.9	0.4
**CAS70**	70.48	14.82	14.7	1st	1148.1	40.5	0.6

4. FEAR simulation of CAS glasses and ring-size quantification

We simulated the structure of three CAS silicate glasses by combining neutron-diffraction experiments and force-enhanced atomic refinement (FEAR) [1]. All simulations were carried out using the Large-scale Atomic/Molecular Massively Parallel Simulator packages [2]. Three CAS glass models, CAS40, CAS50 and CAS70, were computed by molecular-dynamics (MD) simulations with each model comprising around 3000 atoms. We applied the interatomic potential parametrized by Jakse – as it has been found to yield some structural and elastic properties that are in good agreement with experimental data for CAS [3]. A cutoff distance of 8.0 Å was used for the short-range interactions. The Coulombic interactions were calculated by adopting the Fennell damped shifted force model with a damping parameter of 0.25 Å^{-1} and a global cutoff of 8.0 Å. These three glasses were first simulated by MD simulations using a conventional melt-quench method, as described in the following. First, the atoms were randomly placed in a cubic box using PACKMOL

[4] while ensuring the absence of any unrealistic overlap. The systems were then subjected to an energy minimization, followed by some 100 ps relaxations in the canonical (*NVT*) and isothermal-isobaric (*NPT*) ensembles at 300 K, sequentially. These models were then fully melted at 3000 K for 100 ps in the *NVT* and, subsequently, *NPT* ensemble to ensure the loss of the memory of the initial configurations and to equilibrate the system. Then these liquids were cooled from 3000 K to 300 K in the *NPT* ensemble at zero pressure with a cooling rate of 1K/ps. For all simulations, we adopted the Nosé-Hoover thermostat and a fixed time step of 1 fs.

We then assessed the ability of the FEAR [5] (force-enhanced atomic refinement) method to offer an improved description of the atomic structure of glassy silica as compared to those generated by MD or reverse Monte Carlo (RMC). To this end, we adopted the FEAR methodology introduced by Drabold et *al.* [6]. In contrast to MD simulations (which solely uses the knowledge of the interatomic potential) and RMC [7] simulations (which solely uses the knowledge of experimental data), the FEAR approach leverages all the available information. FEAR presents two key advantages: (i) it is more computationally efficient, since the energy does not need to be computed at every RMC step, and (ii) it does not rely on any assumption regarding the weights associated with the structural and energy terms in the cost function. In detail, we first started from a "randomized" structure generated by RMC while using a very high effective temperature, namely, T_χ = 5000 K. Following the original implementation of the FEAR method, the system was then iteratively subjected to a combination of RMC refinements and energy-minimization steps, wherein each FEAR iteration consists of: (i) 3600 RMC steps and (ii) an energy minimization (conducted with the conjugate- gradient method). We found that 16 of such iterations were sufficient to achieve a convergence of the potential energy and R_χ for the CAS glasses. During the refinement, we dynamically adjusted the average acceptance probability of the Metropolis algorithm by linearly decreasing the effective temperature T_χ from 10^2

down to 10^{-3} during the FEAR refinement. These parameters were found to yield a glass structure exhibiting minimum R_χ and potential-energy values.

We then explored the structure of the glass structures generated by FEAR. To this end, we computed the neutron structure factor for each of the simulated glasses. Fig. 5 shows the reduced structure factor, $F(Q)$, predicted by FEAR and MD, which are compared with experimental neutron-diffraction data. We observe that the FEAR-derived structure factors exhibit an excellent agreement with the experimental data over the entire Q range—which is not unexpected, since the neutron PDFs were used as input for the FEAR simulations. In contrast, the MD-derived structure factors present some notable discrepancies with the experimental data. Especially, FEAR predicts a sharper FSDP than MD, suggesting that the glass refined using FEAR exhibits a more ordered medium-range structure than its MD-based counterpart This echoes the fact that glass structures generated by FEAR tend to exhibit an increased thermodynamic stability (i.e., lower energy) as compared to structures generated by MD.

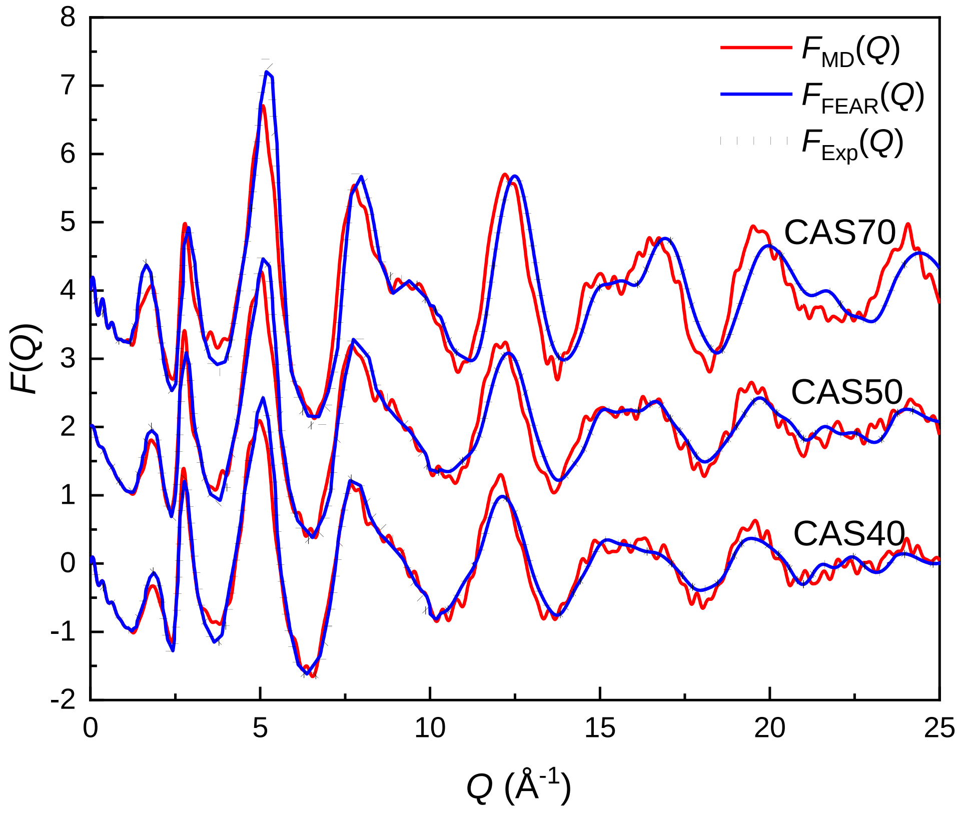

Fig. 5 | Measured (black dotted curves) and two simulated, MD (red solid curves) and FEAR (blue solid curves), total reduced structure factors *F*(*Q*) for three CAS glasses. Each glass *F*(*Q*) is vertically shifted up by 2 for clarity.

We then computed the ring-size distribution from the FEAR-simulated structures using the RINGS code [8]. The Guttman's ring definition was used for ring-counting since it is the most relevant to describe the ring distribution derived from the FSDP of scattering patterns in terms of the probed length scale [1]. The relative ring-size distributions derived from the direct ring counting of the FEAR

structure models were compared with the RingFSDP results and are shown in Fig. 6. There is a significant difference for the percentage of large-sized, ≥6-membered rings between the FEAR and RingFSDP methods. Especially for CAS40 and CAS50 glasses, about 35% of large ≥6-membered rings are counted from the FEAR model while RingFSDP shows no large-sized rings. However, both methods show a similar trend, namely, the fraction of small-sized, ≤4-membered rings significantly and systematically decreases with increasing SiO_2 content.

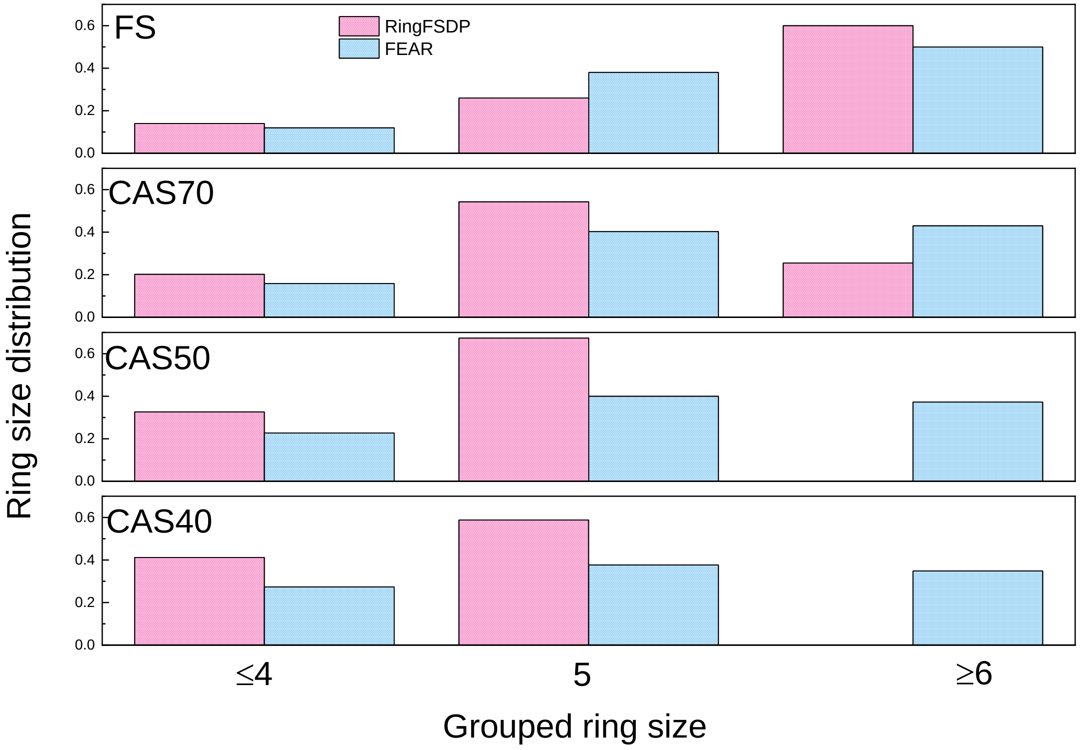

Fig. 6 | Ring-size distribution comparison between the experimental RingFSDP and FEAR simulation analysis.

5. Inaccuracy of DSC-measured fragility data does not affect *m-MRD* correlation

Since it is not possible to obtain and measure every single glass in this study, we also used data from the literature. Unfortunately, some of them had to be calorimetric measurements due to the limited viscosity data in the literature. Considering the possible accuracy

problems in the calorimetric data, we employed the empirical relationship of Zheng et al [24] obtained from the study of various oxide glass systems, including borates, aluminosilicates, and vanadium tellurites. Even though Zheng et al. did not prove that it was a universal empirical relationship, it can be used as a method to improve the accuracy of calorimetric fragility-index data in silicates considering the diversity of oxide glass systems used in that empirical study.

In addition, our goal is to understand and explain the relationship between the structure and the fragility of glasses, but we are not trying to propose a universal empirical model for *MRD* and *m*. Thus, the small inaccuracies in the *m* data do not lead to a change in our observations or the conclusion. In order to demonstrate the effect of the inaccuracy of fragility-index data based on calorimetric measurements, we plot the same dataset without using the correction used in the plot in the manuscript. The comparison of the two plots (Fig. 7) clearly shows that the general trend and correlation between the two parameters do not change when we use the calorimetric data as reported in the cited references without employing the correction. The linear fit quality is slightly worse than the original case, but the difference between the two fits can be considered negligible.

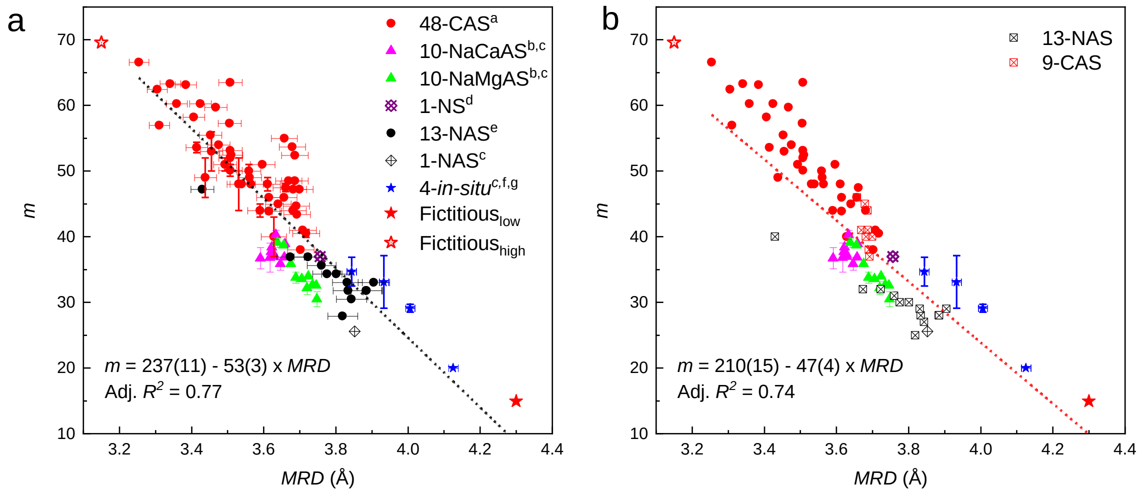

Fig. 7 | Universal inverse linear correlation between *m* and *MRD* values for 88 glasses. The fragility-index values of 9 CAS and 13 NAS glasses from DSC measurements were corrected by Zheng's equation (a) or as reported in the original paper without correction (b).

References

[1] Q. Zhou, Y. Shi, B. Deng, J. Neuefeind, M. Bauchy, "Experimental method to quantify the ring size distribution in silicate glasses and simulation validation thereof," *Sci. Adv.,* vol. 7, p. eabh1761, 2021.

[2] S. Plimpton, "Fast Parallel Algorithms for Short–Range Molecular Dynamics," p.42.

[3] M. Bauchy, "Structural, vibrational, and elastic properties of a calcium aluminosilicate glass from molecular dynamics simulations: The role of the potential," *J. Chem. Phys.,* vol. 141, p. 024507, 2014.

[4] L. Martínez, R. Andrade, E. Birgin, G. Martínez, J. M. PACKMOL, "A package for building initial configurations for molecular dynamics simulations," *J. Comput. Chem.,* vol. 30, pp. 2157-2164, 2009.

[5] Q. Zhou, Y. Shi, B. Deng, T. Du, L. Guo, M.M. Smedskjaer, M. Bauchy, "Revealing the medium-range structure of glassy silica using force-enhanced atomic refinement," *J. Non-Cryst. Solids,* vol. 573, p. 121138, 2021.

[6] A. Pandey, P. Biswas, D.A. Drabold, "Force-enhanced atomic refinement: Structural modeling with interatomic forces in a reverse Monte Carlo approach applied to amorphous Si and SiO_2," *Phys. Rev. B,* vol. 92, p. 155205, 2015.

[7] P. Biswas, R. Atta-Fynn, D.A. Drabold, "Reverse Monte Carlo modeling of amorphous silicon," *Phys. Rev. B,* vol. 69, p. 195207, 2004.

[8] S. Le Roux, P. Jund, "Ring statistics analysis of topological networks: New approach and application to amorphous GeS_2 and SiO_2 systems," *Comput. Mater. Sci.,* vol. 49, pp. 70-83, 2010.

[9] Q. Zheng, J.C. Mauro, Y. Yue, "Reconciling calorimetric and kinetic fragilities of glass-forming liquids," *J. Non-Cryst. Solids,* vol. 456, pp. 95-100, 2017.
